# Puerarin promotes osteogenesis and inhibits adipogenesis *in vitro*

**DOI:** 10.1186/1749-8546-8-17

**Published:** 2013-08-21

**Authors:** Nan Wang, Xinluan Wang, Wenxiang Cheng, Huijuan Cao, Peng Zhang, Ling Qin

**Affiliations:** 1 Translational Medicine R&D Center, Institute of Biomedical and Health Engineering, Shenzhen Institutes of Advanced Technology, Chinese Academy of Sciences, Shenzhen, China; 2 Musculoskeletal Research Laboratory, Department of Orthopaedics & Traumatology, The Chinese University of Hong Kong, Hong Kong SAR, China

## Abstract

**Background:**

Puerarin (daidzein 8-C-glucoside) has potential on preventing osteoporosis. This study aims to investigate the effects of puerarin on osteogenesis and adipogenesis *in vitro*.

**Methods:**

CCK-8 assay, alkaline phosphatase (ALP) activity and Alizarin Red S were used to measure the effects of puerarin on proliferation, osteoblastic differentiation, and mineralization in osteoblast-like MC3T3-E1 cells. The effects of puerarin on adipogenesis were measured by Oil Red O staining and intracellular triglyceride level in preadipocyte 3T3-L1 cells. The mRNA and protein levels of osteogenesis- and adiopogenesis-related factors were detected by qRT-PCR and western blot, respectively. Further, the secreted osteocalcin levels and nuclear translocation of β-catenin were detected by ELISA and immunofluorescence assay, respectively.

**Results:**

As to osteogenesis, puerarin could stimulate proliferation (1 μM, *P* = 0.012; 10 μM, *P* = 0.015; 20 μM, *P* = 0.050), ALP activity (20 μM, *P* = 0.008) and calcium nodule formation (20 μM, *P* = 0.011) in a dose-dependent manner. Puerarin (20 μM) promoted osteocalcin secretion (*P* = 0.004) and the protein expression of both osteopontin (*P* = 0.001) and osteoprotegerin (*P* = 0.003). As to adipogenesis, puerarin suppressed adipocytes formation and intracellular triglyceride level (*P* = 0.001). In addition, puerarin (20 μM) decreased the mRNA and protein levels of CCAAT/enhancer binding protein α (*P* = 0.001, *P* = 0.002), proliferator-activated receptor γ (*P* = 0.005, *P* = 0.003), and adipocyte lipid-binding protein 4 (*P* = 0.001, *P* = 0.001). Moreover, phosphorylation of AKT1-Ser^437^ (10 μM, *P* = 0.003; 20 μM, *P* = 0.007) and GSK-Ser^9^ (10 μM, *P* = 0.005; 20 μM, *P* = 0.003), and the nuclear translocation of β-catenin (10 μM, *P* = 0.006; 10 μM, *P* = 0.002) were increased in 3T3-L1 cells treated by puerarin.

**Conclusion:**

Puerarin promoted osteogenesis and inhibited adipogenesis *in vivo*, and Akt/GSK-3β/β-catenin signaling pathway was involved in the suppression of adipogenesis.

## Background

Postmenopausal osteoporosis is usually associated with aging and decline in gonadal function [1]. The main clinical manifestations of this metabolic disorder are fragility fracture because of imbalance between osteoclast-mediated bone resorption and osteoblast-mediated bone formation [2]. The first-line therapeutic strategy for postmenopausal osteoporosis is estrogen replacement therapy (ERT) to prevent bone loss and increase bone formation [3], through the enhancement of osteoblast differentiation and bone formation [4-6], and inhibition of osteoclast maturation and function [7]. As ERT increases the risk of breast cancer, endometrial cancer and vaginal bleeding [8]. Thus, there are increasing interests in the use of plant-derived estrogens, known as phytoestrogens.

**Figure 1:**
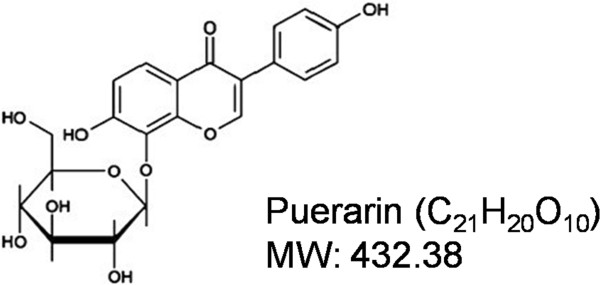
**Chemical structure of puerarin.** Its molecular formula is C_12_H_20_O_10_. MW (molecular weight): 432.38.

Phytoestrogens could bind to estrogen receptors (ERs) and have estrogen-like activity [9]. Puerarin (daidzein 8-C-glucoside) (Figure 1) is one of the major phytoestrogens isolated from the *Pueraria Labata* (Willd.) Ohwi (a wild creeper leguminous plant) [10], which is an important crude herb in traditional Chinese medicine (TCM) for treating various medical conditions, *e.g.*, liver diseases [11], hypertension [12] and angina pectoris [13], as well as a healthy dietary supplement [14]. Recently, researchers tested puerarin on its role in the prevention of osteoporosis, since it could prevent bone loss in ovariectomized (OVX) mice [15] and promote the new bone formation in osteoblast implants [16]. In addition, puerarin significantly increased alkaline phosphatase (ALP) activity and mineral nodules in osteoblast cells [16]. Puerarin also could increase the phosphorylartion of extracellular signal-regulated protein kinase (ERK) and p38-reactivating kinase (p38) to activated MAPK pathway in the endothelial cells [17]. As we know, ERK and p38 are the two main MAPKs which both interplay with BMP pathway in osteogenic differentiation [18]. Mesodermal stem cells (MSCs) can differentiate into a variety of cell types, including osteoblasts, adipocytes, chondroblasts and myoblasts [19], and the osteogenic and adipogenic lineages are closely related [20,21]. When the balance between osteoblast and adipocyte differentiation was disrupted, it would cause diseases, such as osteoporosis or osseous hyperplasia [22,23]. Puerarin and daidzein have similar structures to estrogen. Some reports demonstrated that daidzein promoted osteogenic, inhibited adipogenic differentiation and exhibited preventive activity on bone loss in OVX animals [24,25]. Thus, we would hypothesize that puerarin might promote osteogenesis and inhibit adipogenesis. This study aims to investigate the dual effects and molecular mechanism of puerarin on osteogenesis and adipogenesis *in vitro*.

## Materials and methods

### Materials

Puerarin was purchased from Nanjing TCM institute of Chinese Materia Medica (TCM054-110528, China). The purity of puerarin was 99.35% as tested by high performance liquid chromatography (HPLC) analysis. Cell culture reagents were purchased from Gibco (USA). Cell Counting Kit-8 (CCK-8) was purchased from DOJINDO Lab (Tokyo, Japan). Alkaline phosphatase kit (COD 11592) was purchased from Biosystems (Spain). The primers were purchased from BGI (China). RIPA lysis buffer was purchased from Santa Cruz (CA, USA). BCA protein assay kit (23227), NE-PER nuclear and cytoplastic extraction reagents kit (78833) were purchased from Pierce (USA). RNeasy total RNA extraction kit (AP-MN-MS-RNA-50) was purchasd from Axygen (USA). PrimeScript RT reagent Kit (DRR037A) and SYBR® Green PCR Master Mix (DRR420A) were purchased from TaKaRa (Japan). PVDF membrane was purchased from Bio-Rad (Hercules, CA). ECL advanced western blotting detection kit was purchased from Amersham (UK). Anti-CEBPα antibody, anti-AKT1 antibody, anti-AKT1 (phospho Ser^473^) antibody, anti-GSK3β antibody, anti-GSK3β (phospho Ser^9^) antibody and anti-RUNX2 antibody were purchased from Abcam (USA). PPAR-γ antibody was purchased from cell signaling technology (USA). FABP4 antibody was purchased from ExCell Biology (China). β-catenin rabbit monoclonal antibody and HRP goat anti-rabbit IgG antibody were purchased from Abgent (San Diego, USA), and anti-β actin monoclonal antibody and DyLight 488 AffiniPure goat anti-rabbit IgG (H + L) were purchased from EarthOx (San Diego, USA). Enzyme-linked immunosorbent assay kit for osteocalcin was purchased from Uscn (Wu Han, China). EnzyChromTM triglyceride assay kit was purchased from BioAssay Systems (CA, USA). All the other reagents and chemicals were purchased from Sigma-Aldrich, Inc. (USA).

### Cell culture

3T3-L1 preadipocytes (CL-173) and MC3T3-E1 osteoblastic cells (subclone 14, CRL-2594) were purchased from ATCC (American Type Culture Collection, Manassas, VA, USA). 3T3-L1 cells were maintained in DMEM with 10% FBS and MC3T3-E1 were maintained in α-MEM with 10% FBS in incubator with 5% CO_2_ at 37°C.

### Cell Toxicity

Cell viability was assessed by CCK-8 kit. 3T3-L1 preadipocyte cells or MC3T3-E1 cells were seeded at 5000 cells/well in 96-well plates. After 24 h incubation, the cells were treated with puerarin at concentrations of 0, 0.1, 1, 10 and 20 μM. After 48 h, 100 μL medium solution (content 10% CCK-8) was added and incubated at 37°C for 1 h. Finally, absorbance was measured on a microplate reader Synergy4 (PerkinElmer, USA) at 490 nm. The experiment was repeated three times.

### Bromodeoxyuridine (BrdU) proliferation assay

Cell proliferation was evaluated by the BrdU assay (Roche Applied Science, Germany), a colorimetric immunoassay based on the incorporation of BrdU during DNA synthesis in proliferating cells. Results were obtained according to the manufacturer's instructions by a microplate reader Synergy4 at 490 nm.

### Alkaline phosphatase (ALP) activity assay

MC3T3-E1 cells were plated at a density of 10^4^ cells/well in 24-well tissue culture plates in the growth medium and were cultured until reaching confluence, where the medium was changed to the differentiation medium containing 10 mM β-glycerol phosphate and 50 μg/mL ascorbic acid (regarded as day 0). After another period of 6 days, the medium was removed and the tissue was washed twice with PBS. DEA lysis buffer (100 μL) was added to each well and the plate was shook for 15 min at room temperature. The supernatant was collected after centrifuging at 1400 g rpm for 5 min (eppendorf centrifuge 5417R, Germany). Alkaline phosphatase (ALP) activity was then assayed by a commercial kit. The sample (30 μL) was added to 170 μL of ALP working reagents and the mixture incubated for 5 min. The optical density at 405 nm was measured. The total cell protein was measured by the Bradford's method [26] and the results expressed in nanomoles of p-nitrophenol produced per min per mg of protein.

### Calcium nodule formation

MC3T3-E1 cells (10^5^ cells/well) were seeded in 6-well tissue culture plates in the growth medium for reaching confluence, then continued to incubate in a differentiation medium containing puerarin at 10^-6^, 10^-5^, 2 × 10^-5^ M. On day 8, the cultures in the plates were fixed with 75% ethanol and stained for calcium with 1% Alizalin red S. The stained samples were observed under a dissecting microscope Leica DMI3009B (Germany) and photographed. The amount of calcium deposition was quantified by destaining with 10% cetylpyridinium chloride monohydrate in 10 mM sodium phosphate at room temperature for 15 min. The absorbance was measured at 562 nm.

### Enzyme-linked immunosorbent assay (ELISA)

After osteogenic induction for 6 days, osteocalcin (OC) in the supernatants was directly measured by Enzyme-linked immunosorbent assay kit according to the manufacturer's instructions using a microplate reader Synergy4 (PerkinElmer, USA) at 450 nm. The assays were performed in triplicate and the limit of detection for these immunoassays was 4000 pg/mL according to the manufacturer’s protocol.

### Adipocyte differentiation and Oil Red O staining in 3T3-L1 cells

For adipogenesis, 3T3-L1 cells (5 × 10^4^ cells/well) were plated into a 6-well plate and maintained for 2 days after reaching confluence (designated as day 0). Media were exchanged with differentiation medium (DMEM containing 10% FBS, 0.5 mM IBMX, 1 μM dexamethasone, 2 μg/mL insulin, and 200 μM indomethacin) for 2 days. The cells were then incubated in adipocyte growth medium (DMEM supplemented with 10% FBS and 1 μg/mL insulin) until day 8. Puerarin (10 and 20 μM) and vehicle DMSO were added into the medium over the full course of differentiation. Medium was changed every other day. On day 8, the cells were stained with Oil Red O staining, an indicator of cell lipid content, and digitalized by a Leica microscope DMI3009B (Germany) for analysis.

### Measurement of intracellular triglyceride content

Cells were washed with PBS and solubilized in 5% Triton X-100. The total triglycerides in the lysates were measured by a commercial triglyceride assay kit, according to the manufacturer’s protocol.

### Quantitative Real-time PCR

Total RNA was isolated using the RNeasy total RNA extraction kit from Axygen, following the manufacturer’s protocol. The total RNA (500 ng) was reverse-transcribed to cDNA by PrimeScript RT reagent Kit with oligodT primer and random 6 mers, following the manufacturer’s protocol. The real time PCR primers used in the experiments were shown in Table 1. The final reaction solution (20 μL) contained 1 μL of the diluted cDNA product, 10 μL of 2X Power SYBR® Green PCR Master Mix, 0.8 μL each of forward and reverse primers and 7.4 μL nuclease-free water. The amplification conditions were: 50°C for 2 min, 95°C for 10 min, 40 cycles of 95°C for 15 sec, 60°C for 1 min. The fluorescence signal emitted was collected by Roche LightCycler 480 Detection System (Germany). The mRNA levels of all genes were normalized by β-actin as internal control. These analyses were performed in duplicates for each sample using cells from three different cultures, and each experiment was repeated three times.

**Table 1:** Primer sequences used for real-time PCR

**Gene**	**Forward primer**	**Reverse primer**
M-β-actin	TGTCCACCTTCCAGCAGATGT	AGCTCAGTAACAGTCCGCCTAGA
M-C/ebpα	GAACAGCAACGAGTACCGGGTA	GCCATGGCCTTGACCAAGGAG
M-Pparγ	CGCTGATGCACTGCCTATGA	AGAGGTCCACAGAGCTGATTCC
M-Fabp4	CATGGCCAAGCCCAACAT	CGCCCAGTTTGAAGGAAATC
M-Lpl	GGGAGT TTGGCTCCAGAGTTT	TGTGTCTTCAGGGGTCCTTAG

### Western blotting

The proteins from nucleus and cytoplasm were extracted separately by NE-PER nuclear and cytoplastic extraction reagents kit. Cell pellets were lysed in RIPA lysis buffer with 1% PMSF, 1% protease inhibitor cocktail, and 1% sodium orthovanadate. After treatment on ice for 30 min, cell lysates were clarified by centrifugation at 11,419 g for 30 min at 4°C to remove cell debris, and the protein content was measured by a BCA protein assay kit. Aliquots of the lysates were subjected to 10% SDS-PAGE (with 5% stacking gel) and transferred to a PVDF membrane. The membrane was probed with monoclonal or polyconal antibody (mAb) followed by horseradish peroxidase-conjugated secondary antibodies and visualized by an ECL advanced western blotting detection kit according to the manufacturer’s protocol. β-actin was used as a reference to normalize the differences in the amounts of protein between samples.

### Immunofluorescence assay

After culturing 3T3-L1 cell in differentiation medium for 3 days, the cells fixed with 80% ethanol for 10 min, and incubated with the PBS containing 0.5% Triton X-100, and washed three times with PBS. Cells were incubated with mouse monoclonal antibody (mAb) against β-catenin (1: 50) for 2 h at room temperature, followed by incubation with anti-mouse IgG Alexa 488 antibody (1: 50) for 1 h. After washing with PBS for three times, the cells were incubated for 1 min with DAPI (0.1 μg/mL) for nuclear staining at room temperature. Finally, the cells were examined and photographed by a confocal laser scanning microscope (Leica TCS SP5, Germany).

### Statistical analysis

All quantitative data were presented as means ± standard deviation (SD) of three measurements. Statistical comparisons were performed by the SPSS 17.0 software (Chicago, IL, USA). One-way analysis of variance (ANOVA) followed by Tukey post-*hoc* test (multi-group comparison) was used to assess statistical significance at *P* < 0.05.

## Results

### Puerarin promoted osteogenesis in osteoblast-like MC3T3-E1 cells

**Figure 2:**
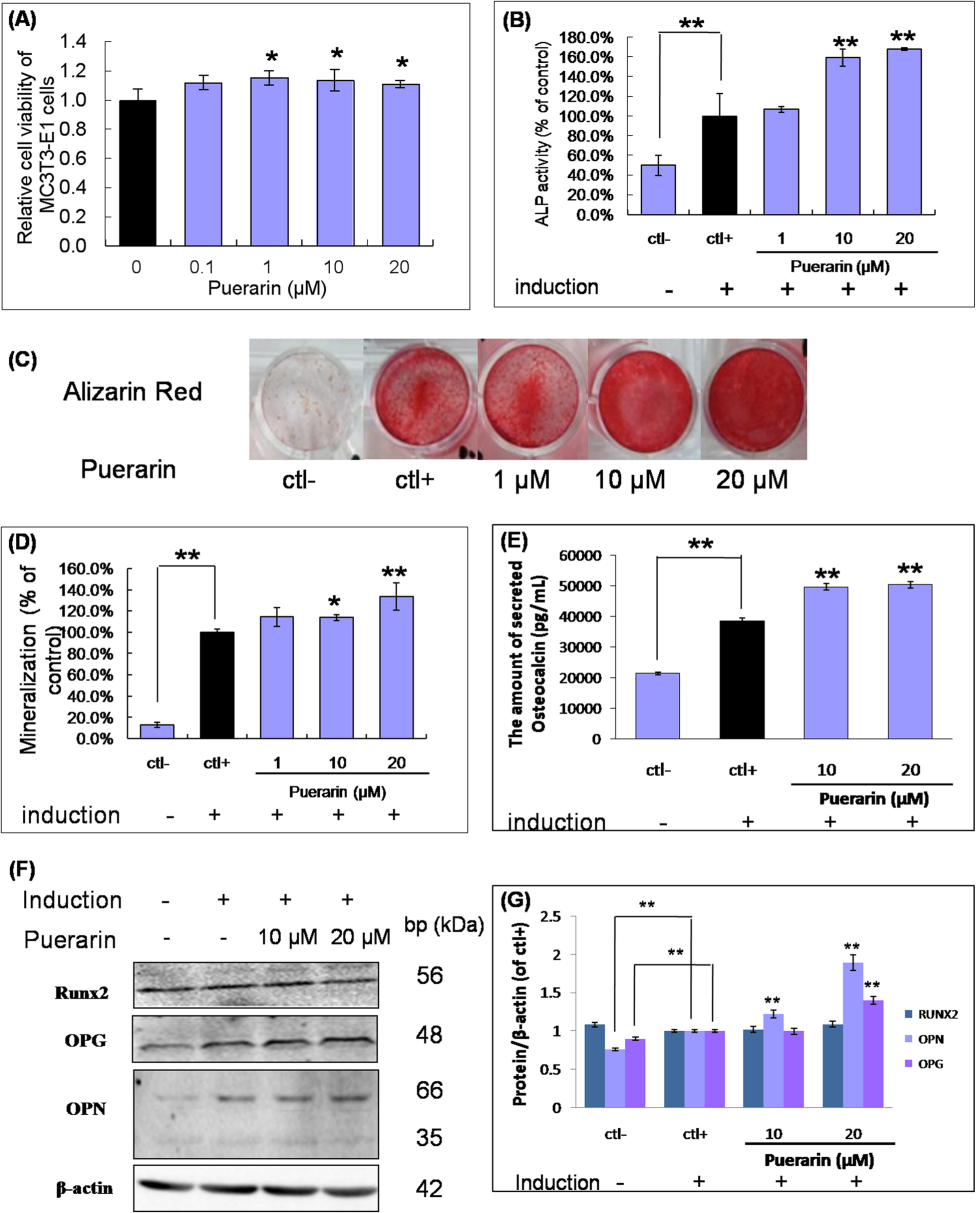
**Puerarin promoted osteogenesis in osteoblast-like MC3T3-E1 cells. (A)** Puerarin promoted MC3T3-E1 osteoblastic cells proliferation. The cells were incubated with puerarin at the concentration of 0.1, 1, 10 and 20 μM for 48 hours before CCK-8 assay. DMSO was served as control. **(B)** Effect of puerarin on ALP activity of MC3T3-E1 cells. MC3T3-E1 cells were cultured with vehicle or various concentrations of puerarin for 6 days. The data are expressed as percentage of positive control (ctl+) that was induced by osteoblastic differentiation. **(C)** Effect of puerarin on the mineralization of extracellular matrix by MC3T3-E1 cells. AR-S staining was performed for the demonstration of mineralized nodule formation at days 8. **(D)** AS-R was then eluted from the matrix and measured by spectrophotometry at 562 nm. **(E)** Secreted Osteocalcin (OC) levels in the media were also measured after 6-day induction. **(F-G)** The protein expression of RUNX2, OPN, OPG was detected by western blot assay and quantification of immunoblots. * *P* < 0.05, ** *P* < 0.01 compared with the cells without treatment by puerarin (n = 3).

MC3T3-E1 osteoblastic cells were cultured in puerarin at various concentrations (0, 0.1, 1, 10 and 20 μM) for 48 h. As shown in Figure 2A, those treated with puerarin promoted MC3T3-E1 osteoblastic cells proliferation at concentrations of 1 (*P* = 0.012), 10 (*P* = 0.015) and 20 μM (*P* = 0.050). As shown in Figure 2B, puerarin demonstrated a dose-dependent effect by visually determined on promoting ALP activity. It significantly increased the ALP activity by 59.3% at the concentrations of 20 μM, compared to the control group (*P* = 0.008). Calcium nodule formation was examined by Alizarin Red S staining. Figure 2C and 2D show that puerarin increased mineralized nodule formation in a dose-dependent manner by visually determined, where the maximal and significant effects were observed at a concentration of 20 μM (*P* = 0.011). Compared with the control group without osteogenic induction (ctl-), the induction group (ctl+) significantly increased OC levels expression. Puerarin further increased the OC levels expression to 36.4% at 10 μM dosage (*P* = 0.003), and 42.2% at 20 μM dosage (*P* = 0.004), compared with ctl+ group (Figure 2E). These results suggested that puerarin could promote proliferation, ALP activity, mineralization and OC protein secretion in MC3T3-E1 cells.

### The effects of puerarin on osteogenesis-related protein expression during osteogenesis differentiation in MC3T3-E1 cells

Runt-related transcription factor 2 (RUNX2) is a key transcription factor in osteoblastic differentiation [27]. We tested whether puerarin could stimulate osteogenesis by modifying the expression of this transcription factor. Osteoprotegerin (OPG) and osteopontin (OPN) required for the differentiation of pre-osteoblasts into mature osteoblast were also detected. As shown in Figure 2F and 2G, the protein of RUNX2 between non-induced and induced group were weak, and the treatment groups with puerarin did not significantly increase the expression of RUNX2 at the sixth day after induction. However, significant increases in OPG (*P* = 0.003) and OPN (*P* = 0.001) protein expression were observed when compared with induction group at 20 μM dosage. These results suggested that puerarin up-regulated the expression of OPG and OPN might contribute to osteoblastic differentiation.

### The effects of puerarin on cell toxicity and BrdU proliferation assay in 3T3-L1 cells

**Figure 3:**
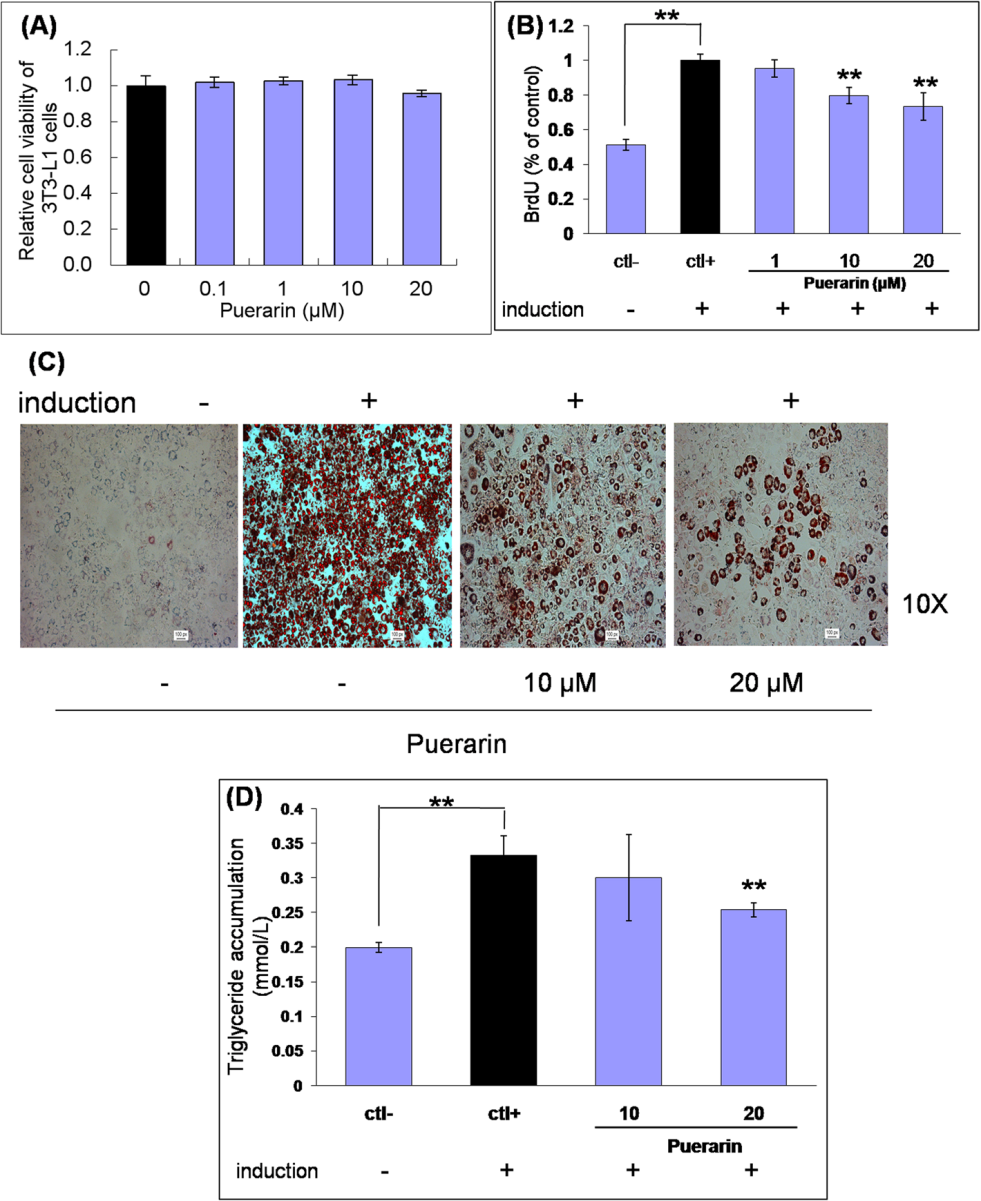
**Puerarin inhibites adipogenesis in 3T3-L1 cells without cell toxicity. (A)** Puerarin had no toxic effect on 3T3-L1 preadipocyte cells on the tested concentrations. **(B)** Puerarin inhibited postconfluent mitotic clonal expansion in 3T3-L1 preadipocytes after 48 hour treatment. **(C)** Puerarin inhibited adipogenesis-induced accumulation of lipids in 3T3-L1 preadipocytes at day 8 after adipogenic induction. Representative morphological changes of 3T3-L1 adipocyte differentiation were by Oil Red O staining. **(D)** Puerarin suppressed triglyceride accumulation during adipogenesis.

As shown in Figure 3A, 3T3-L1 treated with puerarin for 48 h at the selected concentrations (0.1, 1, 10 and 20 μM) did not differ from that of the control group, suggesting that puerarin had no toxic effects on 3T3-L1 preadipocyte cells on 0.1, 1, 10 and 20 μM. At an early stage of differentiation, 3T3-L1 cells proceed through two cycles of mitotic division under adipogenic differentiation [28]. Postconfluent 3T3-L1 cells were cultured in induction medium for 48 h with various doses of puerarin (1, 10 and 20 μM) and measured BrdU at 48 h later. As shown in Figure 3B, puerarin inhibited postconfluent mitotic clonal expansion of 3T3-L1 preadipocytes at early stage of differentiation in a dose-dependent manner.

### The effects of puerarin on adipogenesis in 3T3-L1 cells

After adipogenic differentiation for 8 days, significantly more lipid droplets were observed in adipocyte control cells, as compared with the non-induced cells. However, lipid accumulation was significantly inhibited by the treatment with 10 and 20 μM puerarin in a dose-dependent manner by visually determined (Figure 3C and 3D), suggesting that puerarin could reduce the adipogenesis in 3T3-L1 cells.

### Puerarin decreased gene and protein of expression of adipogenic transcription and adipocyte-specific factors

**Figure 4:**
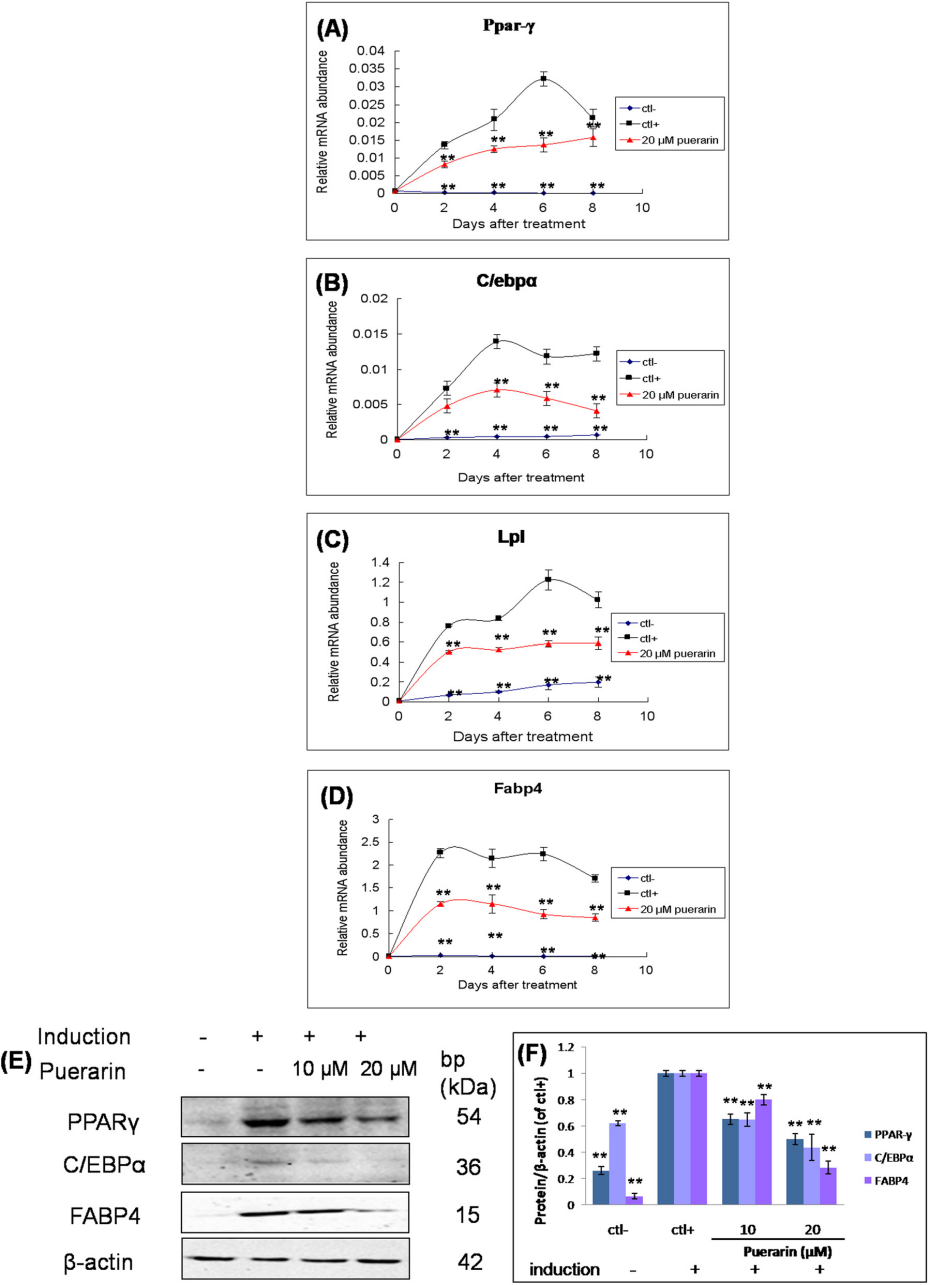
**Puerarin inhibits adipogenesis of 3T3-L1 cells adipocyte differentiation.** mRNA expression levels of **(A)** Ppar-γ, **(B)** C/ebpα, **(C)** Lpl and **(D)** Fabp4 at day 2, 4, 6 and 8 were isolated and detected. **(E-F)** Related proteins (PPAR-γ, C/EBPα and FABP4) of adipogenesis were detected by western blot assay and quantification of immunoblots. * *P* < 0.05, ** *P* < 0.01 compared with ctl+ that was induced by adipogenic induction but without treatment by puerarin at the same time.

During adipocyte differentiation processes, we isolated RNA at day 2, 4, 6 and 8 and detected the changes of selected genes (Figure 4). Two transcription factors, CCAAT/enhancer-binding protein α (C/ebpα) and peroxisome proliferator-activated receptor γ (Ppar-γ) were required for adipocyte differentiation process [29]. Compared to the cells without adipogenic induction, Ppar-γ rose significantly in the adipogenic stimulated cells from day 2 after treatment, and increased sharply at day 4 and 6, and then slowed down in the next two days (Figure 4A). C/ebpα had a similar trend with Ppar-γ (Figure 4B). Puerarin (20 μM) significantly down-regulated the mRNA expression of Ppar-γ and C/ebpα at each time point selected. Puerarin decreased the mRNA expression level of Ppar-γ by 39.7% at day 2 (*P* = 0.009), 39.9% at day 4 (*P* = 0.005), 57.5% at day 6 (*P* = 0.006), and 25.5% at day 8 (*P* = 0.005), and C/ebpα by 34.1% at day 2 (*P* = 0.009), 48.9% at day 4 (*P* = 0.008), 50% at day 6 (*P* = 0.006), and 66.2% at day 8 (*P* = 0.001), compared with that in the adipogenic induced cells without puerarin treatment.

The detected changes of expression of Lpl and Fabp4 confirmed that puerarin suppressed adipogenesis. Lpl and Fabp4 rose significantly in the adipogenic stimulated cells from day 2, reached the highest level at day 2 and day 6, respectively. Puerarin (20 μM) significantly decreased the Lpl mRNA level by 33.90% at day 2 (*P* = 0.009), 37.19% at day 4 (*P* = 0.006), 52.30% at day 6 (*P* = 0.007), and 42.25% at day 8 (*P* = 0.005), while decreased Fabp4 mRNA level by 49.23% at day 2 (*P* = 0.008), 46.38% at day 4 (*P* = 0.005), 50.81% at day 6 (*P* = 0.005), and 50.41% at day 8 (*P* = 0.001), compared with that in the cells induced to adipogenic differentiation without puerarin treatment (Figure 4C and D). These results suggested that puerarin inhibited adipogenesis by down-regulating expression of the adipocyte-related genes.

In Western blot analysis, the expression of adipogenic markers, including PPAR-γ, C/EBPα and FABP4, was significantly decreased by puerarin during cell differentiation (Figure 4E and 4F).

### The effects of puerarin on Akt/GSK-3β/β-catenin signaling pathway

**Figure 5:**
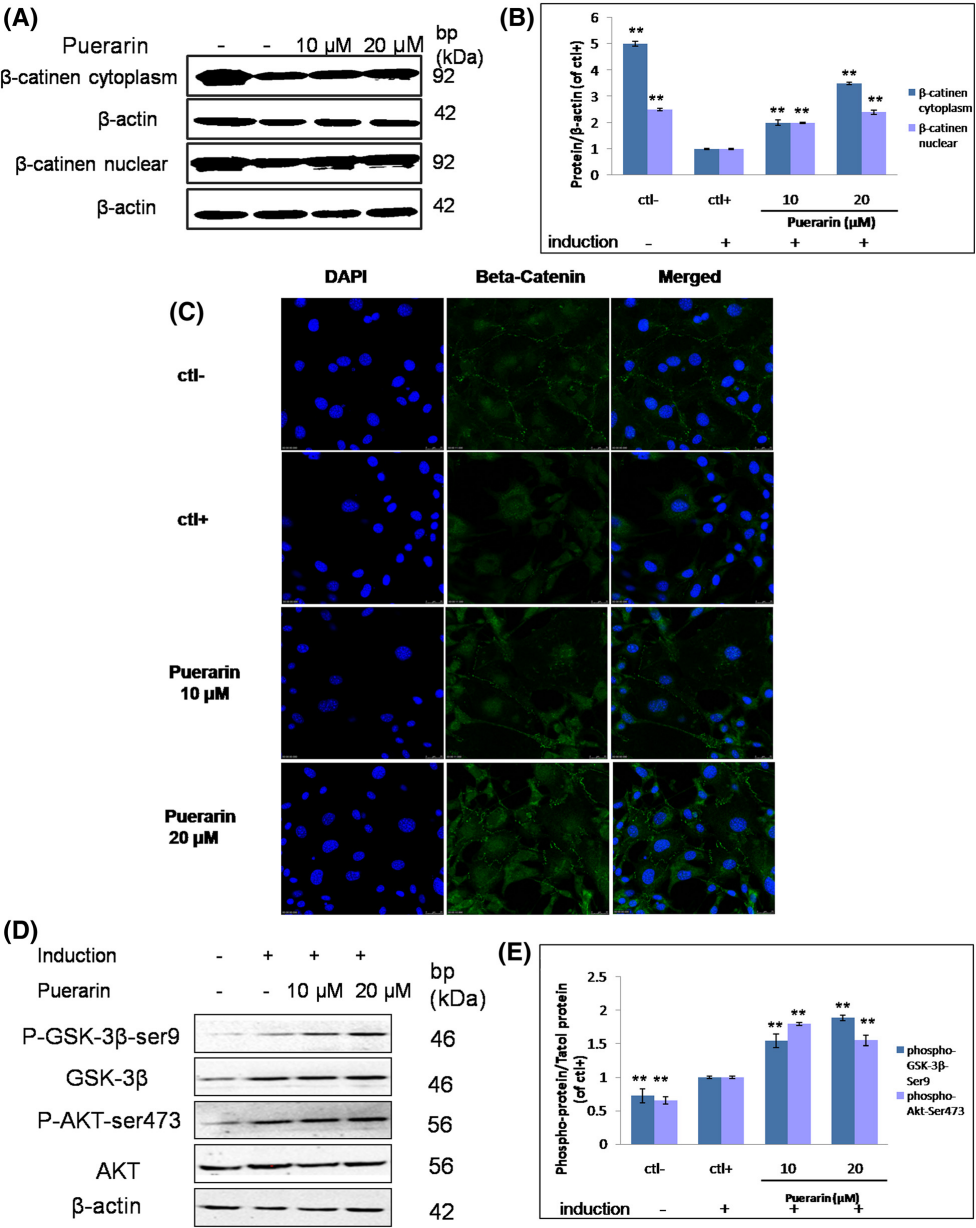
**The effects of puerarin on Akt/GSK-3β/β-catenin signaling pathway. (A-B)** Effects of puerarin on β-catenin protein expression in cytoplasm and nuclear in 3T3-L1 cells. The effect of puerarin on β-catenin was determined by western blotting. **(C)** Effects of puerarin on β-catenin translocation in 3T3-L1 cells. β-catenin expression was labeled with anti-β-catenin antibody and an Alex a fluor 488-conjugated secondary antibody (green). Magnification × 400. **(D-E)** The protein of AKT1, AKT1 (phosphor-Ser^473^), GSK-3β and GSK3β (phospho-Ser^9^) were detected by western blot assay and quantification of immunoblots.

Relatively strong expression of β-catenin were observed in the control groups (cytoplasm and nuclear extraction) and the protein expressions decreased significantly in adipogenic-stimulated group (*P* = 0.0001, *P* = 0.004) (Figure 5A and 5B). Two dosage (10, 20 μM) of puerarin showed similar increased effects (10 μM, *P* = 0.006; 20 μM, *P* = 0.002) on β-catenin protein expression, suggesting that the lowered β-catenin expression by adipocyte differentiation was recovered by treatment of puerarin. The translocation of β-catenin was observed by the confocal laser scanning microscope, as a surrogate marker for the Wnt pathway activation. In control group, β-catenin was predominantly localized in the nucleus (Figure 5C). When 3T3-L1 cells were induced in the adipogenic-stimulated group, the translocation of β-catenin into the cytoplasm was observed. In both treatment groups, the immunofluorescent staining β-catenin relocated into nucleus.

The expression of phospho-GSK-3β-Ser^9^ was significantly increased (*P* = 0.009) in induction group. In the presence of puerarin, the rate of phospho-GSK-3β-Ser^9^/total GSK-3β was dramatically increased (10 μM, *P* = 0.005; 20 μM, *P* = 0.003) in 3T3-L1 adipocytes (Figure 5D and 5E). As expected, the rate of phospho-AKT-Ser^473^/total AKT was also dramatically increased (10 μM, *P* = 0.003; 20 μM, *P* = 0.007) by puerarin in 3T3-L1 cells (Figure 5C and 5E). These observations indicated that puerarin induced the phosphorylation of AKT at serine 473 and subsequently activated the phosphorylation of GSK-3β at serine 9, leading to GSK-3β inhibition and Wnt/β-catenin signaling.

## Discussion

The effect of puerarin on promoting the osteoblast bone formation both *in vitro* and *in vivo*[16,30] and bone tissues and bone metabolism in ovariectomized rates [31], was reported. This study demonstrated for the first time that puerarin promotes osteogenesis and inhibits adipogenesis at the same time.

As the reciprocal relationship between adipogenesis and osteogenesis in the bone marrow would lead to osteoporosis or osseous hyperplasia [22,23], promotion of osteogenesis and suppression of adipogenesis was thought to be an important mechanism for some anti-osteoporotic agents [32]. 3T3-L1 and MC3T3-E1 cell lines were used, because they were the most well-characterized and reliable models for studying the differentiation and functions *in vitro*[33,34].

In this study, phyto-estrogen puerarin enhanced proliferation and differentiation, and also increased the amount of secreted osteocalcin in selected dosage of MC3T3-E1 cells. Meanwhile, puerarin could increase OPG and OPN protein expression, which is consistent with the previous data [35]. The expression profiles of RUNX2 proteins were not significantly different among treatment, non-induction or induction group. This might be due to the factor that RUNX2 is an early transcription factor for osteoblast differentiation [36], while our measurement was carried out for RUNX2 protein at day 6 after osteogenic induction.

During the process of adipogenesis, 3T3-L1 cells proceed through two rounds of cell division as mitotic clonal expansion and then initiate the differentiation program [28]. PPAR-γ is the key transcription factor in adipocytes differentiation and it is expressed at the highest level in adipose tissue and adipocyte cell lines and at low levels, or not at all, in other tissues and cell line [37]. Adipose-specific PPAR-γ knockout mice showed reduced fat mass and protected against high fat diet-induced obesity [38]. PPAR-γ and C/EBPα synergistically activate the downstream promoters of Lpl and Fabp4. During the adipogenesis, puerarin inhibited adipogenesis in 3T3-L1 cells by measuring mitotic clonal expansion at early stage, significantly suppressed PPAR-γ and C/EBPα mRNA and protein levels in the middle stage, and inhibited the mRNA and protein levels of FABP4 in the late stage. These results suggested that puerarin inhibited adipogenesis through down-regulation of PPAR-γ and C/EBPα expression.

Wnt proteins are auto-secreted from cell or adjacent cells, and then act through cell surface receptors to determine the cell fate [39]. Wnt signaling was involved in inhibiting adipogenesis and inducing osteoblastogenesis [33,40,41]. In the canonical Wnt signaling pathway, Fzd signaled through Dishevelled to inhibit the kinase activity of a complex containing glycogen synthase kinase 3 (GSK3), Axin, β-catenin and Adenomatous polyposis coli (APC) [39]. This inhibition facilitated cytosolic β-catenin to accumulate and translocate to the nucleus, where it bounded the TCF/LEF family of transcription factors to regulate the expression of Wnt target genes, such as Runx2, PPAR-γ and C/EBPα, promoting osteogenesis and inhibiting adipogenesis [42-44]. When Wnt signaling was suppressed, this complex targeted β-catenin for phosphorylation to rapid degradation [45]. The protein expression of β-catenin significantly suppressed in adipocyte differentiation group in both cytoplasm and nucleus, while the treatment group with puerarin increased the protein expression in a dose-dependent manner, suggesting that puerarin could increase β-catenin protein expression to inhibit adipocyte differentiation. Fluorescent immunostaining showed that puerarin kept β-catenin protein stay in nuclear in comparison with the induced group. These results confirmed that puerarin exhibited anti-adipogenesis activity probably through the canonical Wnt/β-catenin signaling pathway, which might also facilitate the effect of puerarin on osteogenesis [46].

Wnt/β-catenin and PI3K/Akt/GSK-3β signaling pathway were cross-talked by GSK-3β and β-catenin [47]. In the PI3K/Akt/GSK-3β signaling pathway, Akt regulated adipogenesis *via* the phosphorylation and inactivation of substrates, such as GSK-3β, which directly regulated β-catenin. GSK3β is also a component of the canonical Wnt signaling pathway, controling the activity of β-catenin in the context of a multimolecular complex, *e.g.*, adenomatous polyposis coli (APC) and axin. Thus, activation of Akt is important for anti-adipogenesis, which would activate PPAR-γ and C/EBPα during 3T3-L1 adipocyte differentiation [48,49]. Puerarin increased the rate of phospho-AKT-Ser^473^/total AKT, and phospho-GSK-3β-Ser^9^/total GSK-3β in a dose-dependent manner, which might lead to the cytosolic β-catenin to accumulate and translocate to the nucleus and activation of Wnt/β-catenin signaling pathway.

Isoflavones are structurally similar to estrogen and has estrogen-like activity that is mediated through estrogen receptors (ER) [17]. Estradiol could induce the association of estrogen receptor α (ERα) with insulin-like growth factor-1 (IGF-I) receptor (IGF-IR) and activates the PI3K/Akt/GSK3β signaling pathway [50]. Similar to estrodiol, puerarin was also reported to activate ER-dependent PI3K/Akt pathway in the endothelial cells [17], and it might lead the following action of puerarin: increasing the amount of active Akt (ser-473 phosphorylate), GSK3β (ser-9 phosphorylate) and stabilizing and accumulating β-catenin in nucleus, which might result in the dual effects of puerarin on promoting osteogeneis and suppressing adiopogenesis. Puerarin improved insulin sensitivity and deceased total cholesterol in serum from rats fed a high-fat diet [51,52], and markedly improved insulin resistance of 3T3-L1 lipocyte by suppressed PPAR-γ mRNA expression and promoted Glut-4 transposition to cell membrane to increase the transportation of glucose [53].

In postmenopausal osteoporosis, the decreased number of osteoblasts may be due to increased differentiation of the BMSCs to the adipogenic lineage. Puerarin indeed promoted nuclear translocation of β-catenin, which might play an important role in promotion of osteogenesis and inhibition of adipogenesis.

## Conclusion

Puerarin promoted osteogenesis and inhibited adipogenesis *in vitro*, and Akt/GSK-3β/β-catenin signaling pathway was involved in the suppression of adipogenesis.

## Abbreviations

ALP: Alkaline phosphatase; APC: Adenomatous polyposis coli; BrdU: Bromodeoxyuridine; CCK-8: Cell counting kit-8; C/EBPα: CCAAT/enhancer binding protein α; ERK: Extracellular signal-regulated kinase; ELISA: Enzyme-linked immunosorbent assay; ERT: Estrogen replacement therapy; ER: Estrogen receptor; Fabp4: Adipocyte lipid-binding protein 4; FZD: Frizzled; GSK3: Glycogen synthase kinase 3; HPLC: High performance liquid chromatography; IGF-I: Insulin-like growth factor-1; IGF-IR: IGF-I receptor; IR: Insulin resistance; Lpl: Lipoprotein lipase; LRP: LDL-receptor-related protein; MAPK: Mitogen-activated protein kinase; MSCs: Mesodermal stem cells; OC: Osteocalcin; OPG: Osteoprotegerin; OPN: Osteopontin; OVX: Ovariectomized; PPAR-γ: Proliferator-activated receptor γ; RUNX2: Runt-related transcription factor 2; TCM: Traditional Chinese medicine.

## Competing interests

All authors declare that they have no competing interests.

## Authors’ contributions

XLW and LQ designed the study. NW performed the experiments and data analysis. NW and XLW, WXC, HJC and PZ interpreted the data and wrote the manuscript. All authors read and approved the final version of the manuscript.
